# Gut microbiota differs between ICU patients admitted for cardiac arrest and other causes: a secondary, propensity-matched cohort analysis

**DOI:** 10.1186/s40635-025-00803-2

**Published:** 2025-08-28

**Authors:** Hannah Wozniak, Eleonora Balzani, Vladimir Lazarevic, Nadia Gaia, Aude de Watteville, Raphaël Giraud, Jacques Schrenzel, Claudia Heidegger, The Gut Microbiota Working Group, Hannah Wozniak, Lorin Fröhlich, Tania Soccorsi, Noémie Suh, Aurélie Perret, Chiraz Chaabane

**Affiliations:** 1 Intensive Care Unit, Department of Acute Medicine, Geneva University Hospitals, Geneva, Switzerland; 2 Centre for Medical Sciences-CISMed, University of Trento, Via S. Maria Maddalena 1, 38122 Trento, Italy; 3 Genomic Research Laboratory, Department of Medicine, Geneva University, Geneva, Switzerland

**Keywords:** Gut microbiota, Critical illness, Cardiac arrest

## Abstract

**Background:**

Critical illness is known to reduce gut microbiota (GM) diversity, a change associated with adverse outcomes. Among potential mechanisms, splanchnic hypoperfusion may play a key role. Cardiac arrest (CA), characterized by transient global hypoperfusion, provides a relevant model to explore this effect.

**Results:**

We conducted a secondary, propensity score-matched analysis of a cohort study investigating GM changes during early intensive care unit stay. Stool samples were collected at ICU admission (S1) and at least 24 h later (S2). GM profiling was performed using 16S rRNA sequencing. Shannon diversity index and taxonomic composition were compared between CA and non-CA patients. Propensity score matching and generalized linear models (GLM) were used to adjust for confounding. A total of 26 patients were included in this analysis (13 CA, 13 matched controls). At S1, CA patients had significantly lower GM diversity (Shannon index: 3.6 [3.0–3.8] vs. 4.3 [3.9–4.8], *p* = 0.019). This was confirmed in the GLM (*β* = − 0.30, SE 0.12, *p* = 0.022). At S2, diversity remained lower (3.2 [2.7–3.8] vs. 4.0 [3.7–4.3], *p* = 0.064). While no global compositional shifts were observed between groups, differences in the abundance of specific taxa were noted.

**Conclusion:**

CA is associated with reduced GM diversity in the first few days of intensive care unit admission compared to non-CA patients, supporting a role for splanchnic hypoperfusion in GM modulation. Further research should investigate clinical consequences and evaluate microbiota-targeted interventions in this high-risk population.

**Supplementary Information:**

The online version contains supplementary material available at 10.1186/s40635-025-00803-2.

## Background

Gut microbiota (GM) is recognized as a dynamic and integral component of human health and has gained increasing attention in recent research due to its essential role in host immunity, metabolism, and homeostasis [1, 2].

Critical illness has been associated with significant disruptions in commensal GM, characterized by a loss of microbial diversity and an increased predominance of potentially pathogenic bacteria—a phenomenon referred to as dysbiosis [3]. Several key findings have emerged from recent research [3–10]. First, GM diversity and richness decline rapidly after intensive care unit (ICU) admission, and this early reduction has been found to be associated with adverse outcomes, including increased mortality in some studies [7–9]. Second, GM composition appears to exhibit distinct signatures depending on the underlying disease process [9, 11–13]. Third, multiple factors contribute to dysbiosis, including the critical disease itself, a decrease in the gastrointestinal transit time [14], drug administration (e.g., antibiotics, proton pump inhibitors, norepinephrine) [15–17], and changes in nutritional intake [18]. Another potential mechanism is splanchnic hypoperfusion, which can lead to tissue hypoxia, disrupted mucus production, and enterocyte injury, which in turn promote cell apoptosis and pathogen translocation [1, 19, 20]. However, the relative contribution of each factor and the specific role of splanchnic hypoperfusion remain uncertain.

Some indirect evidence supports the role of hypoperfusion in the development of dysbiosis. In healthy individuals, even transient hypoperfusion from intense physical exertion, such as 60 min of cycling, increases intestinal permeability [21]. In heart failure, splanchnic hypoperfusion causes ischemia and intestinal edema, facilitating bacterial translocation and the entry of bacterial metabolites into circulation via a compromised gut barrier [22]. Lastly, in patients with cardiac arrest (CA), biomarkers of intestinal injury are elevated, and these are associated with endotoxemia, suggesting that gut translocation contributes to its development [23, 24].

To better assess the potential impact of splanchnic hypoperfusion on GM, an ideal model would be patients who experience a period of hypoperfusion, such as those with CA. Indeed, CA is a situation where transient and global splanchnic ischemia occurs for at least one hour after the return of spontaneous circulation [25]. In a previous study, our group assessed GM changes in critically ill patients and found that early reduction in GM diversity was associated with increased mortality [7]. If splanchnic hypoperfusion is indeed a key driver of GM alterations, then patients with the most pronounced hypoperfusion—CA patients—should exhibit the greatest microbial shifts. Importantly, our hypothesis centers on the impact of hypoperfusion during the CA event itself, which occurs prior to ICU admission and prior to sample collection. Therefore, we hypothesize that even if hemodynamic parameters are normalized by the time of stool sampling, the initial ischemic insult may have already led to early and detectable shifts in GM.

Therefore, we conducted a secondary analysis of our existing dataset, comparing GM changes between CA patients and those admitted to the ICU for other reasons. We aim to provide the first description of GM in CA patients and gain further insight into the possible role of splanchnic hypoperfusion in shaping GM alterations in ICU patients.

## Method

### Study design and patients

This is a secondary analysis of a previously conducted single-center prospective cohort study that described GM changes during the first days of ICU admission and their association with mortality [7]. The original study included adult ICU patients admitted for various critical illnesses, excluding those with recent antibiotic use, chronic gastrointestinal diseases, or immunosuppression. Fecal samples were collected prospectively and consisted of two time points: (1) the first spontaneously passed stool after ICU admission, and (2) a second stool passed at least 24 h later. Standardized procedures were used for collection and storage. Consent was obtained from the patient or a surrogate decision-maker in accordance with institutional guidelines. Retrospective consent was sought for patients who regained decision-making capacity. The study was approved by the Geneva Ethical and Research Committee (BASEC-2021-00315) and conducted in accordance with the Declaration of Helsinki.

Further details on inclusion criteria, patient characteristics, and primary findings have been described elsewhere [7].

### Gut microbiota analysis

Details on the GM analysis and sequencing methods are available elsewhere [7]. For this study, we conducted a derivative analysis based on the relative abundances of zero-radius operational taxonomic units (zOTUs), zOTU richness, and the Shannon diversity index, as generated in the original study [7]. These variables were calculated from fecal samples collected at two time points: the first stool passed after ICU admission (S1) and a second sample (S2), collected at least 24 h later.

To assess the significance of differences in bacterial communities between groups defined by the reason for ICU admission, we performed a PERMANOVA [26] test with 9999 permutations using PRIMER (PRIMER-e, Auckland, New Zealand). This test was based on Bray–Curtis similarity [27]. To explore variations in overall bacterial communities, principal coordinate analysis (PCoA) was performed (PRIMER) using a Bray–Curtis similarity matrix built from square root-transformed relative abundances of zOTUs.

### Statistical analysis

To account for potential confounders and ensure comparability, we applied propensity score matching using the nearest neighbor matching method. The propensity score was estimated through a logistic regression model based on age, Charlson comorbidity index, and the absence of antibiotic administration prior to the first stool collection. After estimating the propensity scores, matching was performed in a 1:1 ratio without replacement to balance baseline characteristics between groups.

To assess covariate balance before and after matching, standardized mean differences (SMDs) were calculated and visualized using Love plots, with an absolute SMD < 0.1 considered indicative of good balance.

Continuous variables were described as medians with interquartile ranges (IQR), while categorical variables were presented as counts and percentages (*n*, %). Comparisons between groups were performed using the Wilcoxon rank-sum test for continuous variables and Fisher’s exact test for categorical variables. To assess the relative abundance of taxa in relation to D-60 survival status, a MaAsLin3 [28] algorithm was used applying the abundance model, log transformation, and a minimum prevalence of 20%.

A generalized linear model was then used to assess the association between CA and GM diversity (Shannon index) while adjusting for residual confounding. All statistical analyses were performed using R (v4.1.3, R Foundation for Statistical Computing, Vienna, Austria; URL: https://www.R-project.org).

## Results

### Baseline characteristics of ICU patients with and without cardiac arrest

A total of 26 patients were included in the analysis, with 13 patients admitted for CA and 13 admitted for other reasons. One CA patient died before the second stool collection (Table 1).

**Table 1:** Baseline characteristics of ICU patients with and without cardiac arrest

Characteristic	Overall (*N* = 26)	No cardiac arrest (*N* = 13)	Cardiac arrest (*N* = 13)	*p*-value
Age (years) (median [IQR])	59.5 [45.0–74.0]	67.0 [45.0–74.0]	57.0 [54.0–74.0]	0.9
Sex male (*n*, %)	14 (54%)	9 (69%)	5 (38%)	0.12
Charlson comorbidity index (median [IQR])	4 [1–5]	4 [3–5]	3 [0–5]	0.7
Type 2 diabetes, *n* (%)	8 (26.9%)	4 (30.8%)	3 (23.1%)	> 0.9
History of a cerebrovascular accident, *n* (%)	3 (11.5%)	1 (7.7%)	2 (15.4%)	> 0.9
Peripheral vascular disease, *n* (%)	5 (19.2%)	1 (7.7%)	4 (30.8%)	0.32
History of myocardial infarction, *n* (%)	1 (3.4%)	0 (0%)	1 (7.7%)	> 0.9
Chronic heart failure, *n* (%)	3 (11.5%)	1 (7.7%)	2 (15.4%)	> 0.9
Reason for ICU admission (*n*, %)				
Sepsis	3 (21.5%)	2 (15.4%)	1 (7.7%)	0.4
Cardiovascular disease	5 (19.2%)	1 (7.7%)	4 (30.8%)	
Respiratory insufficiency	8 (30.8%)	3 (23.1%)	5 (38.5%)	
Neurological disease	6 (23.1%)	4 (30.8%)	2 (15.4%)	
Other^1^	4 (15.4%)	3 (23.1%)	1 (7.7%)	
APACHE II Score at ICU admission (median [IQR])	23.0 [20.0–28.0]	23.0 [21.0–27.0]	22.0 [20.0–28.0]	0.6
SAPS II Score at ICU admission (median [IQR])	53.5 [43.0–71.0]	56.0 [53.0–71.0]	43.0 [33.0–70.0]	0.061
Mechanical ventilation (*n*, %)	26 (100%)	13 (100%)	13 (100%)	> 0.9
Mechanical ventilation (days) (median [IQR])	10.0 [9.0–19.0]	12.5 [10.0–19.5]	10.0 [6.0–14.0]	0.3
Cardiac assistance (*n*, %)	3 (12%)	2 (15%)	1 (7.7%)	> 0.9
Noradrenaline on admission	23 (88%)	11 (85%)	12 (92%)	> 0.9
Noradrenaline at S1 (*n* %)	23 (88%)	11 (85%)	12 (92%)	> 0.9
Noradrenaline at S2 (*n* %)	16 (70%)	8 (73%)	8 (67%)	> 0.9
Days of noradrenaline (median [IQR])	6.0 [2.0–14.0]	6.0 [4.0–14.0]	5.5 [2.0–15.0]	0.7
State of shock^2^ on ICU admission (*n*, %)	11 (44%)	4 (33%)	7 (54%)	0.3
Lactate on ICU admission (median [IQR])	2.1 [1.6–3.6]	2.1 [1–2.9]	2 [1.7–3.6]	0.83
Lactate at S1, (median [IQR])	1 [0.8–1.7]	1 [0.8–1.2]	1.1 [0.8–1.8]	0.83
Lactate at S2, (median [IQR])	1 [0.8–1.4]	1 [0.8–1.2]	0.9 [0.7–1.6]	0.86
SOFA score at admission (median [IQR])	10.0 [8.0, 13.0]	9.0 [6.0, 11.0]	11.0 [10.0, 13.0]	0.15
SOFA at S1^3^ (median [IQR])	8 [5–10]	8 [7–10]	9 [4–10]	0.5
SOFA at S2^3^ (median [IQR])	6 [4–9]	6.5 [5–8]	6 [4–9]	> 0.9
Antibiotic between ICU admission and S1	26 (100%)	13 (100%)	13 (100%)	> 0.9
Antibiotic between S1 and S2	23 (88%)	13 (100%)	10 (77%)	0.2
ICU length of stay, days (median [IQR])	17.0 [10.0–31.0]	21.0 [15.0–45.0]	13.0 [9.0–29.0]	0.2
Hospital length of stay, days (median [IQR])	38.5 [18.0–78.0]	52.0 [31.0–124.0]	35.0 [16.0–60.0]	0.069
60-day mortality (*n*, %)	7 (27%)	3 (23%)	4 (31%)	> 0.9
Time to S1, days from ICU admission (median [IQR])	3 [2, 3]	3 [2, 3]	3 [2, 3]	> 0.9
Time between S1 and S2, days (median [IQR])	3 [2–6]	4 [2–7]	3 [2, 3]	0.7
Shannon index at S1, zOTU (median [IQR])	3.9 [3.6–4.4]	4.3 [3.9–4.8]	3.6 [3.0–3.8]	0.019
Shannon index at S2, zOTU (median [IQR])	3.7 [3.0–4.1]	4.0 [3.7–4.3]	3.2 [2.7–3.8]	0.064

^1^Metabolic disorders, kidney failure, abdominal disease, drug abuse, ear–nose–throat surgery

^2^*Shock state was defined as MAP < 65 mmHg and/or lactate > 2 mmol/L and/or vasopressor use

^3^S1: first stool sample collected after ICU admission; S2: second stool sample collected at least 24 h after S1

Covariate balance between groups was assessed using a Love plot and showed adequate balance after matching (Supplementary Fig. 1).

**Fig. 1:**
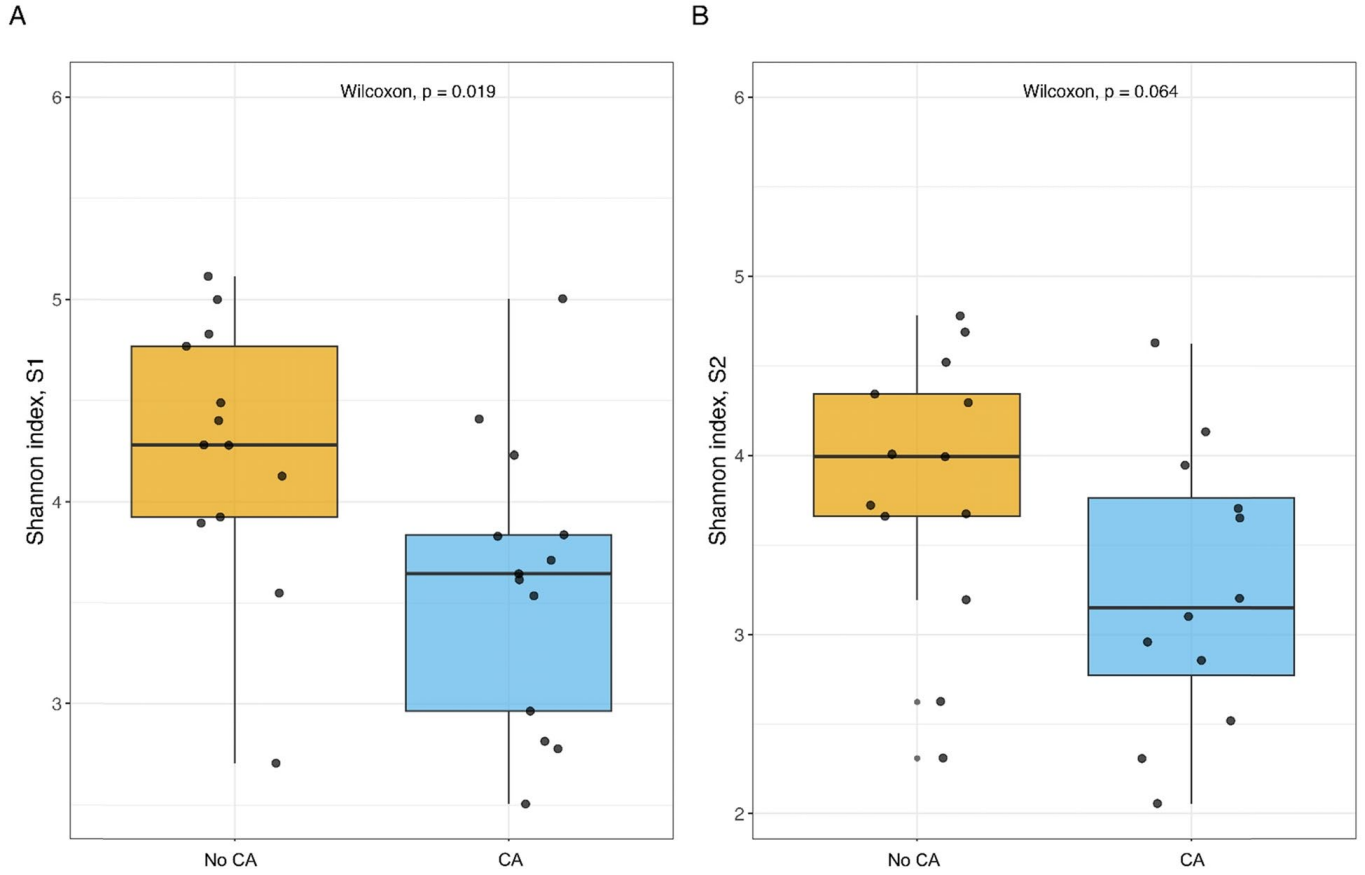
Comparison of Shannon diversity index Between ICU patients with and without cardiac arrest. Each dot represents an individual patient’s microbiota diversity. **A** Shannon diversity index at S1. Patients with cardiac arrest (CA) had a significantly lower Shannon index compared to non-CA patients (Wilcoxon *p* = 0.019). **B** Shannon diversity index at S2. The difference between groups was no longer statistically significant but showed a similar signal (Wilcoxon *p* = 0.064)

At ICU admission, the Shannon diversity index at the zOTU level was significantly lower in CA patients compared to non-CA patients (3.6 [3.0, 3.8] vs. 4.3 [3.9, 4.8], *p* = 0.019). A similar trend was observed at S2 (3.2 [2.7, 3.8] vs. 4.0 [3.7, 4.3], *p* = 0.064), although the difference did not reach statistical significance (Table 1, Fig. 1). Likewise, OTU richness showed a trend towards reduced values in CA patients, but this difference was not statistically significant (Supplementary Fig. 2).

Regarding severity scores, APACHE II and SAPS II scores at ICU admission were similar between groups (APACHE II: 22.0 [20.0, 28.0] vs. 23.0 [21.0, 27.0], *p* = 0.6; SAPS II: 43.0 [33.0, 70.0] vs. 56.0 [53.0, 71.0], *p* = 0.061). Mortality at day-60 did not differ significantly between groups (31% vs. 23%, *p* > 0.9).

**Fig. 2:**
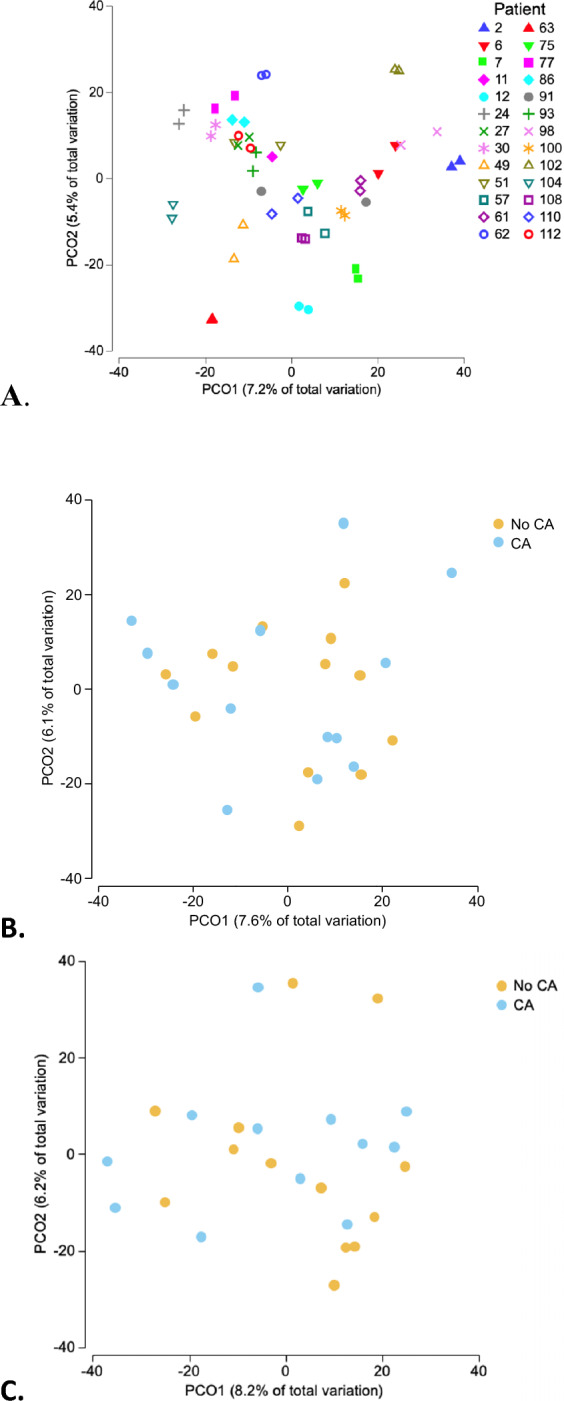
Principal coordinates analysis (PCoA) of gut microbiota composition in ICU patients. **A** PCoA plot colored by individual patient, showing that microbiota profiles primarily cluster by patient, with no major overall changes. **B** PCoA at S1, stratified by CA vs. non-CA status, showing no distinct separation between groups. **C** PCoA at S2, stratified by CA vs. non-CA status, also showing no global shift in microbiota composition between groups

PCoA analysis showed that microbiota profiles primarily clustered by individual patient (Fig. 2A). At S1 and S2, there was no clear separation between CA and non-CA patients, indicating no major global shift in gut microbiota composition between groups (Fig. 2B and C). However, numerous taxa exhibited differential abundance (MaAsLin 2) between CA and non-CA patients (Supplementary Fig. 3). PERMANOVA analysis confirmed that GM composition significantly differed between patients (*p* = 0.0001), indicating strong inter-individual variability.

### Generalized linear model for Shannon diversity index in ICU patients with and without cardiac arrest

A generalized linear model was performed to assess the association between CA and GM diversity (Table 2).

**Table 2:** Generalized linear model for Shannon index in ICU patients with and without cardiac arrest

Shannon index S1	Estimate (*β*)	Standard error (SE)	*P* value
Cardiac arrest	− 0.30	0.12	0.022
Shannon index S2	Estimate (*β*)	Standard error (SE)	*P* value
Cardiac arrest	− 0.23	0.12	0.073

At S1, the Shannon diversity index at the zOTU level was significantly lower in CA patients (*β* = − 0.30, SE 0.12, *p* = 0.022). At S2, a similar trend was observed (*β* = − 0.23, SE 0.12, *p* = 0.073).

## Discussion

In this secondary analysis of a single-center prospective cohort study, we found that patients admitted to the ICU for cardiac arrest had lower gut microbiota diversity at ICU admission, as reflected by a lower Shannon index in the first stool sample, compared to those admitted to the ICU for other reasons. This trend persisted over time, although the difference was no longer statistically significant in later samples, possibly due to limited statistical power.

Previous research has identified trimethylamine N-oxide (TMAO), a metabolite produced through gut microbiota metabolism, as a secondary biomarker of intestinal dysbiosis, with higher TMAO levels associated with increased mortality in CA patients [29]. Although GM alterations are known to influence TMAO levels, no study has previously described GM composition and its changes in CA patients compared to non-CA ICU patients. By demonstrating lower GM diversity in CA patients, we provide new insights into this specific population and further evidence that splanchnic hypoperfusion might disturb the GM. Since reduced GM diversity has been associated with infections, organ failure, and mortality in critically ill patients [5, 7–9], identifying CA patients as particularly vulnerable to it is crucial. Further research is needed to determine whether the extent of dysbiosis in CA patients correlates with important clinical outcomes in this specific patient population, including neurological recovery, secondary infections, and mortality.

Interestingly, while GM diversity was altered, we did not observe a significant shift in overall microbiota composition between CA and non-CA patients. This suggests that although diversity is affected, the broad structure of the GM remains relatively stable across ICU patients. Given this observation, interventions aimed at restoring microbiota diversity, such as probiotics or prebiotics, may be more relevant than approaches targeting specific bacterial taxa.

This study has several limitations. First, the small sample size and monocentric design may limit generalizability and reduce statistical power to detect subtle microbiota changes. Second, as a secondary analysis, it is subject to the constraints of the original study design. Specifically, data on the timing and duration of the CA were not available. This limits our ability to differentiate between distinct clinical subtypes of CA (e.g., in-hospital vs. out-of-hospital, short vs. prolonged arrest), which may be associated with varying degrees of hypoperfusion and gut injury. Third, while we observed differences in microbiota diversity, PERMANOVA analysis showed strong inter-individual variability (*p* = 0.0001), suggesting that baseline microbiota composition influences GM dynamics during critical illness. Future studies should account for this variability to better assess ICU-related effects. In addition, while Shannon diversity is a widely used metric, it may not fully capture compositional shifts. To address this, we also reported relative abundance of zOTUs that significantly differed between CA and no-CA patients. Several zOTUs showed differences in relative abundance between CA and non-CA patients, but these taxa were generally of low abundance, and none remained statistically significant after correction for multiple testing. These findings are therefore exploratory and should be interpreted with caution. Fourth, although longitudinal changes in microbiota diversity between S1 and S2 may offer prognostic insights, we did not explore this due to the limited sample size and the risk arising from multiple testing. Fifth, while 16S rRNA sequencing is a widely used and validated approach for characterizing microbial community structure, it does not allow for strain-level resolution and does not provide information on microbial metabolic functions. Future studies may benefit from integrating broader sequencing approaches (e.g., shotgun metagenomics) and metabolomic profiling. Finally, while our findings support the hypothesis that splanchnic hypoperfusion contributes to gut dysbiosis, this remains hypothesis-generating. Future studies should consider integrating candidate markers of splanchnic hypoperfusion, such as intestinal fatty acid-binding protein (I-FABP), or metabolites associated with gut dysbiosis, such as TMAO [23, 29], while acknowledging that their validation as objective indicators remains incomplete. This has the potential to help identify high-risk patients and guide targeted interventions to mitigate the impact of dysbiosis in critically ill populations.

## Conclusion

Cardiac arrest patients exhibit a gut microbiota profile with lower diversity. However, the overall microbial structure remains unchanged, suggesting that CA primarily affects microbial diversity rather than community composition. Future research should investigate how this loss of diversity relates to clinical outcomes in broader cohorts and whether microbiota-targeted interventions could benefit these patients. Functional characterization through metagenomic and metabolomic approaches may provide deeper mechanistic insight.

## Supplementary Information

Supplementary Material 1

## Data Availability

The datasets used and/or analyzed during the current study are available from the corresponding author on reasonable request.

## Abbreviations

GM: Gut microbiota
CA: Cardiac arrest
ICU: Intensive care unit
zOTUs: Zero-radius operational taxonomic units
