# Supplementary material for: Gut microbiota differs between ICU patients admitted for cardiac arrest and other causes: a secondary, propensity-matched cohort analysis
Source: Intensive Care Med Exp. 2025 Aug 28;13:88. doi: 10.1186/s40635-025-00803-2 (PMC12394093; doi:10.1186/s40635-025-00803-2)
Supplement: Supplementary file 1 — Supplementary Material 1 [file 40635_2025_803_MOESM1_ESM.docx]

**Supplementary Appendix**

**Gut Microbiota Differs Between ICU Patients Admitted for Cardiac Arrest and Other Causes: A Secondary, Propensity-Matched Cohort Analysis**

Table of contents

Supplementary Fig. 1..…………………………………………………………………….…2

Supplementary Fig. 2..…………………………………………………………………….…3

Supplementary Fig. 3..…………………………………………………………………….…4

**Supplementary Figure 1. Love Plot Showing Covariate Balance (Nearest Neighbor Matching)**


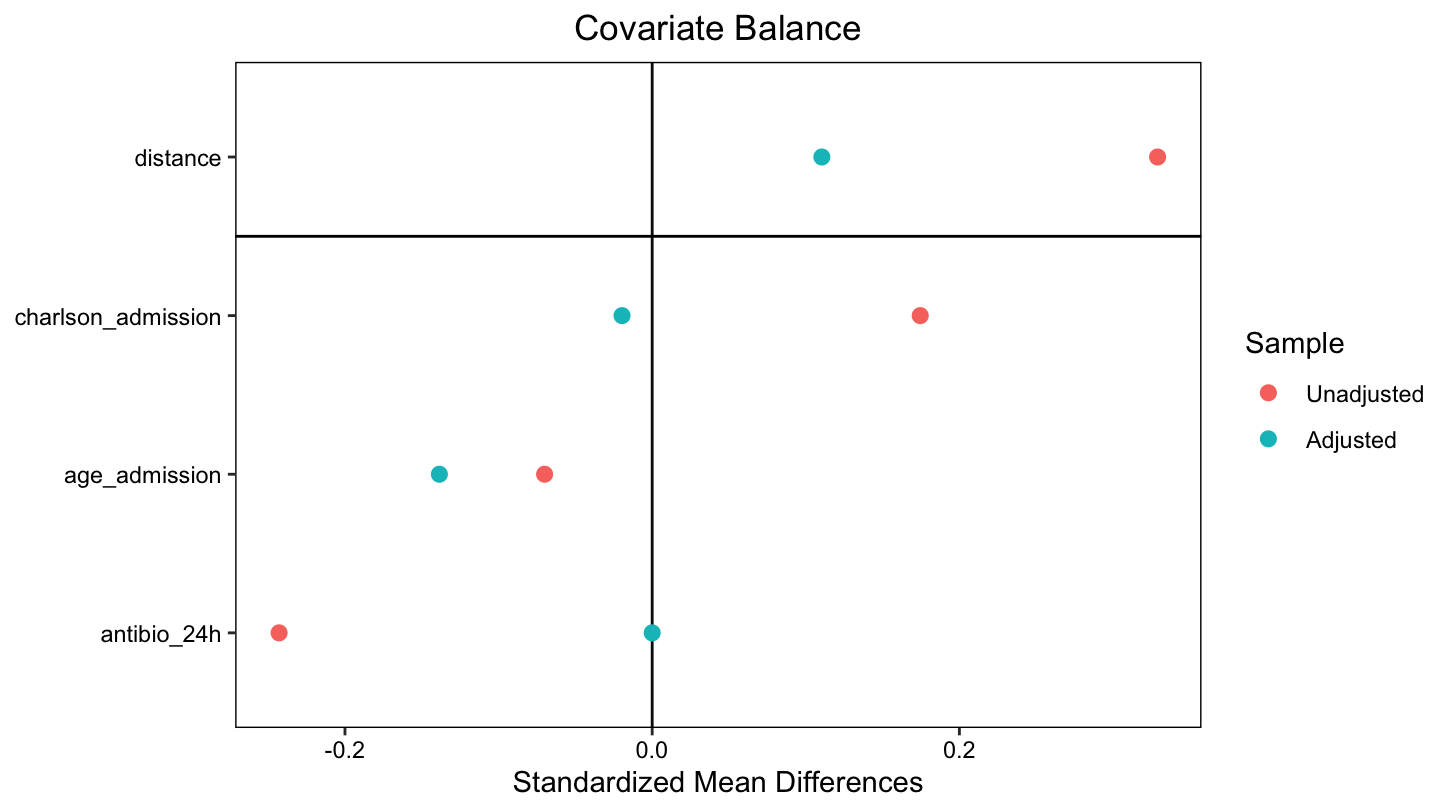


**Supplementary Figure 2.** Comparison of Microbial Richness (zOTU Count) Between ICU Patients With and Without Cardiac Arrest

**
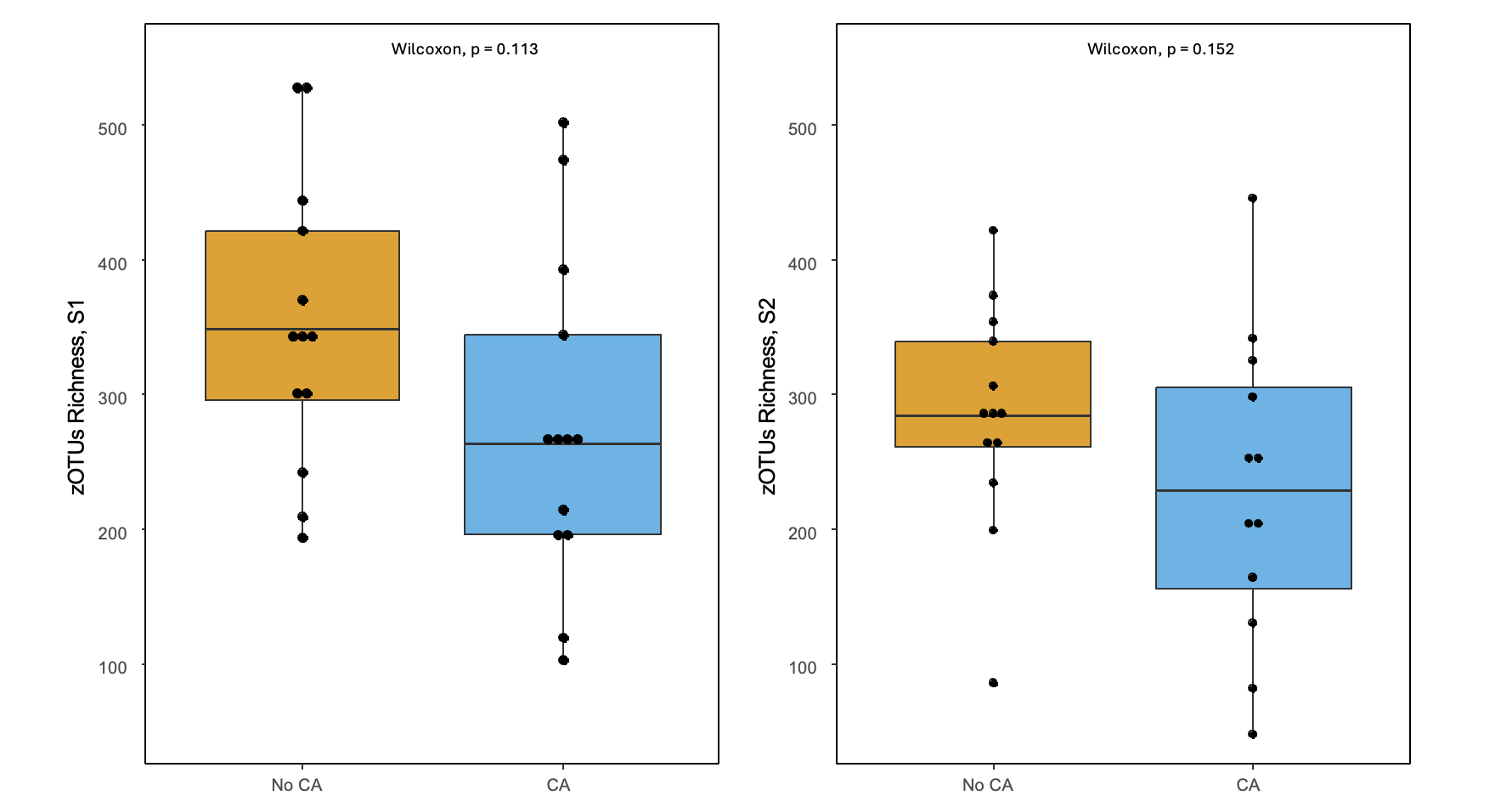
**

**Figure Legend:** Each dot represents an individual patient’s microbial richness, expressed as the number of observed zOTUs (zero-radius Operational Taxonomic Units).
Panel (A): Microbial richness at S1. Patients with cardiac arrest (CA) had a lower richness compared to non-CA patients, but the difference was not statistically significant (Wilcoxon p = 0.113). Panel (B): Microbial richness at S2. A similar trend was observed, with lower richness in the CA group, though again not reaching statistical significance (Wilcoxon p = 0.152).

**Supplementary Figure 3. Differentially abundant bacterial taxa between cardiac arrest (CA) and non-cardiac arrest (No CA) patients at ICU admission (S1) and after at least 24 hours (S2).**

A.
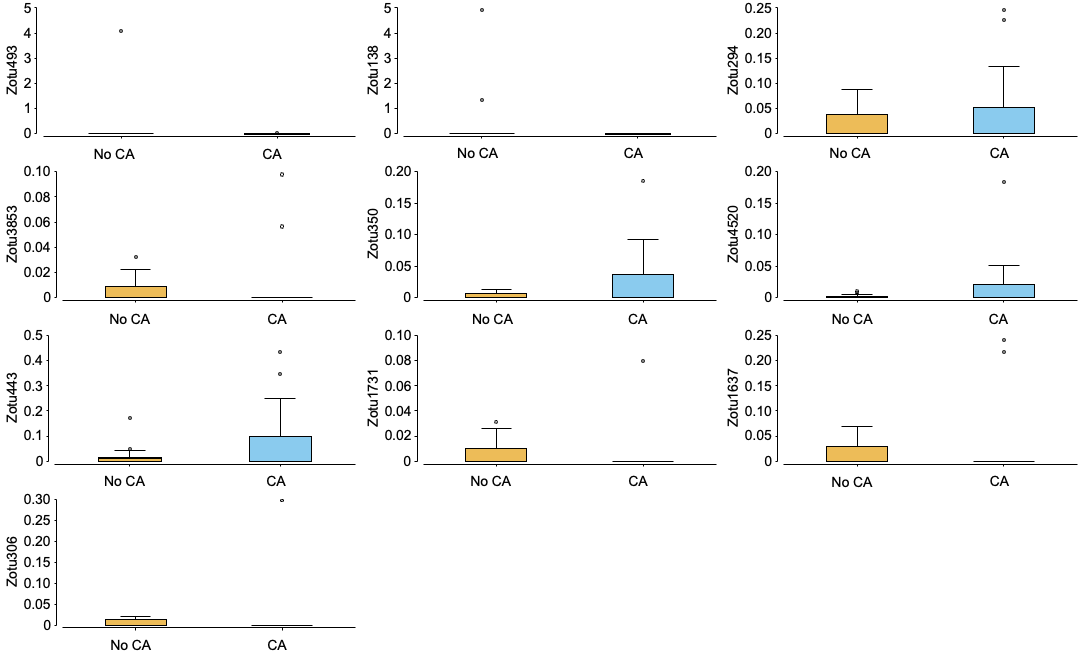


B.
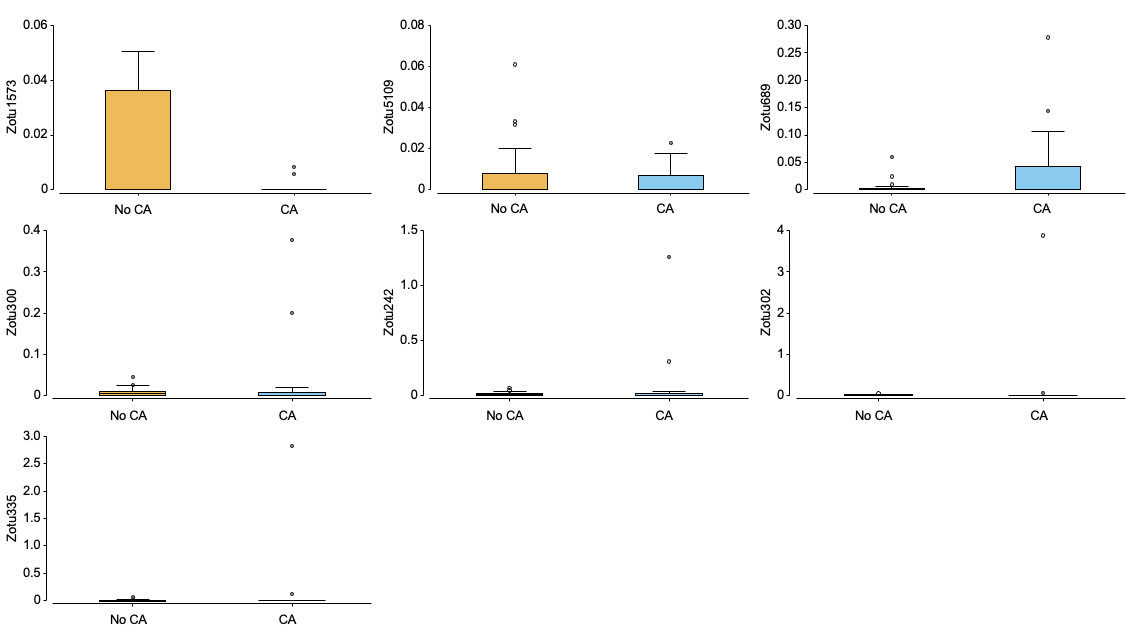


Figure Legend: Boxplots represent the relative abundance of zOTUs that significantly differed between CA and no-CA patients, as identified using MaAsLin2 (without corrected p-values).

(A) S1: Zotu493 (*Bacteroides uniformis*), Zotu138 (*Enterococcus durans*), Zotu294 (*Alistipes shahii*), Zotu3853 (*Merdimonas faecis*), Zotu350 (*Bacteroides fragilis*), Zotu4520 (FMEU_s), Zotu443 (*Parabacteroides distasonis*), Zotu1731 (*Faecalibacterium* GG697149_s), Zotu1637 (*Faecalibacterium* unclassified), Zotu306 (*Bacteroides* unclassified). (B) S2: Zotu1573 (*Parvimonas micra*), Zotu5109 (*Butyricimonas* PAC001510_s), Zotu689 (*Parabacteroides distasonis*), Zotu300 (*Longicatena* JH590969_s), Zotu242 (*Eggerthella lenta*), Zotu302 (*Bacteroides* unclassified), Zotu335 (*Bacteroides* unclassified).
